# What is it like to live with narcolepsy? A scoping review

**DOI:** 10.1007/s11325-025-03259-6

**Published:** 2025-02-08

**Authors:** Jan Hlodak, Andrea Madarasova Geckova, Simona Carnakovic, Eva Feketeova

**Affiliations:** 1 Comenius University, Faculty of Social and Economic Sciences, Institute of Applied Psychology, Bratislava, Slovakia; 2 University of Pavol Jozef Safarik, Medical faculty, Institute of Health Psychology and Research Methodology, Kosice, Slovakia; 3 University of Pavol Jozef Safarik, Faculty of Medicine, 1st Dept. of Psychiatry, Košice, Slovakia; 4 University Hospital of L. Pasteur, Kosice, Slovakia; 5 University of Pavol Jozef Safarik, Faculty of Medicine, Dept. of Neurology, Košice, Slovakia

**Keywords:** Excessive daytime sleepiness, Narcolepsy, Quality of Life, Health-related quality of life, Scoping review

## Abstract

**Background:**

Narcolepsy impacts quality of life (QoL) with its symptomatology in hobbies and everyday activities, work and productivity and has social and economic consequences. The aim of this review is to map and synthesize evidence about QoL in narcolepsy patients and to focus on research strategies and publications in the matter.

**Methods:**

A scoping review of articles published between 2014–2025. The initial search of WoS resulted in 7748 articles and 2583 in PubMed being screened for eligibility. Intervention, comorbidity, non-narcolepsy, prevalence and medical trials studies were excluded. We extracted data on bibliometric characteristics, research questions, sample and recruitment method, design, concepts and measures, and the main findings. Two independent reviewers did the screening and analyses. The analyzed data were consulted on with stakeholders to settle gaps, possibilities and directions for future research. This study followed the PRISMA-ScR guidelines.

**Results:**

Twenty papers were included in this study. There is an increasing trend in publishing studies focused on QoL in narcolepsy patients, but its spread is very limited across various audiences. Most of the studies assess the association of narcolepsy symptoms, treatment, mental health or nutritional status and QoL in narcolepsy patients. Most used was a questionnaire-based cross-sectional design comparing a control group vs narcolepsy patients recruited through regular follow up at a sleep clinic or national reference centers or patients’ organization.

**Conclusion:**

There is a need to spread knowledge beyond the neurology audience, to widen the scope of research beyond the burden of the symptoms and to employ explorative qualitative designs.

**Supplementary Information:**

The online version contains supplementary material available at 10.1007/s11325-025-03259-6.

## Introduction

Narcolepsy is a rare, lifelong neurological non-progressive sleep-related disorder [1]. According to the International Classification of Sleep Disorders, third edition (ICSD-3), it is classified as one of the central disorders of hypersomnolence. The disease itself manifests by excessive daytime sleepiness (EDS) with both narcolepsy “type one (NT1)” and “type two (NT2)” as the main symptom. The difference between NT1 and NT2 is dependent on the occurrence of cataplexy, which only manifests in NT1, triggered mostly by a strong emotion. Other significant symptoms are sleep paralyses and hallucinations [2, 3]. The prevalence of narcolepsy in American population was 12.6 NT1 cases and 25.1 NT2 cases per 100 000 individuals [4]. According to the European narcolepsy study the mean time of diagnostic delay is 9.7±11.5 years [5]. This condition significantly impacts quality of life because of the effects of EDS and cataplexy on, e.g., hobbies and everyday activities, work and productivity [6, 7] and because it has social and economic consequences [8]. Regime (e.g., lifestyle modification, sleep habits strategies) accompanied by patient adherence and the availability of social support complement pharmacological treatment. As Pérez-Carbonell et al. [9] suggest, poor adherence to medication is visible even in patients with poor symptom control. In their study, only 50.2% of patients showed good adherence to medication. Another challenge might be low awareness not just among public but also among healthcare frontliners [10].

Summing up, narcolepsy is a rare disease affecting QoL, with a diagnostic delay and adherence-related challenges, and we need to understand what the gaps in research are and suggest strategies for improvement. Therefore, our aim is to conduct a scoping review of recent peer-reviewed articles indexed in the Web of Science and PubMed databases. This study aimed to answer the research question “What is already known about QoL in narcolepsy patients?” and the following sub questions: (1) Is there an increasing trend of publication on this topic and how much is the knowledge spread across various audiences? (2) What research questions have already been explored in existing research? (3) What samples and recruitment methods were used in surveys? (4) What study designs were used to answer research questions? (5) What concepts and measurements were used to answer the research questions? (6) What have we learned from these studies about what living with narcolepsy is like? and (7) What are the gaps in research on QoL in narcolepsy patients?

## Methods

This scoping review followed guidelines of the Preferred Reporting Items for Systematic Reviews and Meta-Analyses Extension for Scoping Reviews (PRISMA-ScR). This extension is adapted from the original PRISMA statement and contains 22 checklist items [11].

### Research strategy

Our methodological approach is also based on the six steps of conducting a scoping review [12]. We identified a team of researchers and professionals and selected a specific role (searching and selecting for articles; analyzing; expert supervision, etc.). After identification of the research questions, we focused on a literature search.

### Step 1: identifying the research question

A preliminary search of Web of Science (WoS) and PubMed resulted in finding over 10,000 research papers, in which we did not find any scoping review research article. Therefore, we chose this methodology to find out when and where were the studies published; what research questions were explored; what sample and requitement strategies were used; what designs, concepts and measures were used; and what is it like to live with narcolepsy based on the results? Additionally, we focused on what are the gaps in current knowledge in the topic according to the studies and consultation with stakeholders. We have also screened for existing reviews and to our knowledge, there is no review article focused specifically on all of the research questions we focused on in this paper.

### Step 2: identifying relevant studies

The main paper screening of WoS and PubMed for this review was performed on October 18^th^, 2024. The initial scheme of searching for the research articles contained the keyword “narcolepsy”, which resulted in finding 10,866 research papers focused on this topic. The further selection process had to be specified to minimize the number of papers and to find only appropriate articles. For full electronic search strategy for PubMed see Appendix 1.

### Step 3: eligibility criteria

**Fig. 1.**
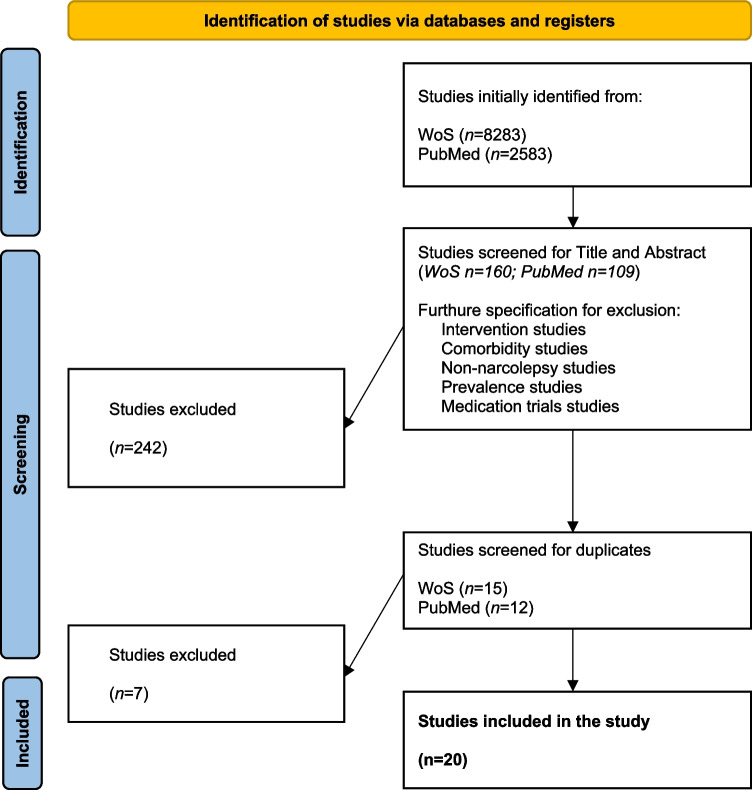
Flow diagram of the searching process and screening strategy

The selection process (see Fig.1) included a specification of article properties: that it be original articles (not reviews) in English with a range of publication dates from 2014 to 2025 and excluding articles about children. The determining terms for further specification were “quality of life; health-related quality of life; mental health”, which reduced the number of research articles to 160 (WoS) and 109 (PubMed).

Next, these articles were then screened according to the titles and abstracts. A total of 242 articles were excluded for not fitting our research questions (articles focused on interventions, comorbidities, non-narcolepsy studies, prevalence and medication trial studies were excluded). Seven duplicates were found. Exclusion process was conducted by two independent reviewers (AMG & JH), and any disagreements were resolved by closer examination of the problematic articles; a final decision was made through discussion, including 20 articles into this review.

### Step 4: charting the data

The research articles used in this scoping review were analyzed independently by two reviewers (JH & AMG). First, we developed a table of variables (a codebook) searched for in the articles based on the first few research papers. Those included basic information about the authors, year of publication, journal name and country of origin. The primary aim was to identify the research questions and their descriptions, year of data collection, study design (e.g., mixed method, cross-sectional etc.) and design category (quantitative/qualitative). In the results we focused on the sample description (e.g., drug naïve/free patients) and the representation of narcolepsy patients (narcolepsy type one and/or type two), sociodemographic indicators (e.g., education level, employment, marital status etc.), recruitment strategies (e.g., mail, clinics, online) and measurement tools (specific questionnaires) focused on measuring sleepiness symptoms, quality of life and other constructs (e.g. depression, anxiety etc.). In the results, we focused on conceptualized variables, statistical and nonstatistical analyses, findings and study limitations. These findings were later compared by the two reviewers, checked for errors or missed information/data, and a final table was created.

### Step 5: collacting, summarizing and reporting the results

The data were collected and summarized as mentioned above. The codebook was later checked by the two reviewers for matchings. These checked coded segments were transformed into tables according to their specificity: the origins of articles and journals; research questions; population, sample and recruitment; and design, concepts and measure. Articles were later described and compared with the results. The results were described to narratively specify the QoL in narcolepsy patients.

### Step 6: consulting stakeholders

These data were later reviewed by a narcolepsy specialist (EF) and a medical doctor (SC) as members of the research team. Stakeholders’ group consisted of two medical doctors (co-authors of this paper), and two other experts in the field of psychology and research. A discussion was held to state gaps in the current research strategies found according to the scoping review. This step is an addition to the PRISMA guideline according to the approach of Mak & Thomas [12]. The discussion was analyzed individually (by JH and AMG) based on the conventional content analysis [71]. Analyzed content was later discussed until no differences were available to consider and a consensus was created. This approach was performed to enrich the findings from the scoping review.

## Results

### When and where were findings about narcolepsy patients’ quality of life published?

There are 20 relevant studies published between 2014 to 2025, only four of which were published in first 5-year period (2014–2019) and the other sixteen in last five years (2020–2024); thus, the prevalence of studies tripled. Most of the studies were published in journals within the WoS category “Clinical Neurology”, but four of them were published in “Psychiatry” or the “Neurosciences” category. Almost a third of them were published in Q1 (n=7) and almost half in Q2 (n=9) journals based on the SCIE relative impact factors for 2022.

### What research questions were explored in published studies?

Most studies compared the HRQoL of narcolepsy patients with control groups, while four studies included comparisons with other diagnostic groups, such as patients with obstructive sleep apnea, insomnia, or idiopathic hypersomnia. Several studies examined associations between narcolepsy symptoms (e.g., symptom severity, disrupted nocturnal sleep, cataplexy), treatment, mental health (e.g., anxiety, depression), and nutritional status. Other topics included psychiatric comorbidities, the prevalence of depressive symptoms, suicidal thoughts, and addictive online behaviors. One study focused on patient resilience, while another explored the impact of narcolepsy on social relationships, including friendships, romantic relationships, and sexual relationships. Two exploratory studies were conducted: one examined how individuals with narcolepsy frame, remember, and report personal experiences with cataplexy, and the other investigated attitudes toward physical health, physiotherapy, and barriers to participation.

### What sample and recruitment methods authors used in their studies?

Eleven studies focused on the European region (France: 2, Italy: 5, Czech Republic, Sweden, Ireland, and the UK). Most collected data over several years, with one longitudinal study examining real-life HRQoL over six years. Recruitment primarily occurred through sleep clinics, though national reference centers and patient organizations were also utilized (e.g., Narcolepsy Centre of Bologna, Italy, in three studies). All studies included NT1 patients, while NT2 patients were included in only some. Two studies focused exclusively on young adults (aged 18–35 or 18–39), and one study examined senior patients (aged 60+). Control groups were drawn from the general population, students, university employees, or respondents’ acquaintances. Sample sizes ranged from 22 to 9,312 narcolepsy patients, with the smallest sample coming from a qualitative study [17] and the largest from the Truven Health Analytics MarketScan Research Databases [38].

### What design, concepts and measures authors used in their studies?

Out of twenty selected studies, eighteen of them were designed as quantitative studies using questionnaire data. Fifteen of these studies were cross-sectional and two were retrospective using data from the routine databases. Two studies used a longitudinal study design, one of them to measure treatment effect as an additional measure to the HRQoL concepts [13], the other to measure real-life changes of HRQoL [14]. Ong et al. [15] also used focus groups; Davidson et al. [16] used open-ended questions together with quantitative data to enrich the findings. All these studies focused on hard data and questionnaire research design. Only two studies were purely qualitative, one of them using a narrative inquiry approach to describe the experiences of only cataplexy symptoms [17] and the other to understand the perception of narcolepsy patients on physical activity and physiotherapy [18].

All the studies set down the specific sociodemographic and anamnestic data used in the research to describe the sample characteristics or for statistical purpose, always stating the sex and age of the respondents’ groups, varying in personal characteristic of education levels, race and ethnicity, employment, geographical region, marital status, family status, etc. They focused on the following diagnosis characteristics: age at onset, age at diagnosis, medication, comorbidities, polysomnography measurements and symptom severity. Most of the studies (n=13) used a specific questionnaire instrument to measure sleep-related symptoms, included the Epworth Sleepiness Scale [19]; the Narcolepsy Severity Scale (n=4) [20] and the Insomnia Severity Index (n=3) [21].

HRQoL was measured with a specific instrument in thirteen studies (out of 18 quantitative studies). The most frequent instrument for measuring QoL was the Health Survey Short Form-36 [22], used in six cases; the European Quality of Life-5 Dimensions Questionnaire [23] used in seven cases; and the PROMIS depression, anxiety, fatigue, sleep disturbances, sleep-related impairment, pain interference, physical functioning [24] in one case together with the SF-36. Other concepts measured in the studies can be derived to mental health and physical health. With mental health, the most common concepts measured were depression and anxiety, usually measured using the Beck Depression Inventory [25] and the Hospital Anxiety and Depression Scale [26]. In one study, addictive online behavior was measured, and in another psychiatric comorbidities were measured, and one focused on cognitive functioning. With social aspects of health, one study [16] focused on social support, social and romantic relationships and communication with others. Kapella et al., [27] also focused on health-related stigma. With physical health, the most common indicator was Body Mass Index (n=9); other concepts were physical fitness and in two studies autonomic dysfunction, one study focused on procrastination and one on coping orientation (see Tables 1, 2, 3, 4).

**Table 1:** Source, year of publication, Web of Science category, SCIE quartiles

		Journal	Year of publication	WoS category	SCIE 2022
1	Kamada et al. [39]: Burden of narcolepsy in Japan: A health claims database study evaluating direct medical costs and comorbidities.	Sleep Medicine	2024	Clinical Neurology	Q1
2	Varallo et al. [37]: Navigating narcolepsy: exploring coping strategies and their associations with quality of life in patients with narcolepsy type 1.	Scientific Reports	2024	Multidisciplinary	Q1
3	Bassi et al. [6]: Work productivity and activity impairment in patients with narcolepsy type 1.	Journal of Sleep Research	2024	Clinical Neurology	Q2
				Neuroscience	Q2
4	Li et al. [34]: The Impact of Symptom Severity on Health-Related Quality of Life in People with Narcolepsy Type 1	Behavioral Sleep Medicine	2023	Clinical Neurology	Q3
				Psychiatry	Q3
5	D’Alterio et al. [32]: Resilience and its correlates in patients with narcolepsy type 1.	Journal of Clinical Sleep Medicine	2023	Clinical Neurology	Q2
6	Tadrous et al. [18]: Exploring exercise, physical wellbeing and the role of physiotherapy: perspectives from people with narcolepsy.	Journal of Sleep Research	2024	Clinical Neurology	Q2
				Neuroscience	Q2
7	Cremaschi et al. [36]: Health-related quality of life in patients with narcolepsy types 1 and 2 from a Sleep Center in Brazil	Arquivos de Neuro-Psiquiatria	2023	Neurosciences	Q4
				Psychiatry	Q4
8	Davidson et al. [16]: The impact of narcolepsy on social relationships in young adults	Journal of Clinical Sleep Medicine	2022	Clinical Neurology	Q2
9	Cambron-Mellot et al. [29]: Examining the impact of excessive daytime sleepiness on utility scores in patients with obstructive sleep apnoea and/or narcolepsy in five European countries.	BMC Neurology	2022	Clinical Neurology	Q2
10	Chin et al. [14]: Quality of life changes and their predictors in young adult narcolepsy patients after treatment: A real-world cohort study.	Frontiers in Psychiatry	2022	Psychiatry	Q2
11	Barateau et al. [33]: Linking clinical complaints and objective measures of disrupted nighttime sleep in narcolepsy type 1	Sleep	2022	Clinical Neurology	Q1
				Neurosciences	Q1
12	Varallo et al. [35]: Exploring Addictive Online Behaviors in Patients with Narcolepsy Type 1	Healthcare	2022	Health Policy & Service	Q2
				Health Care Science & Services	Q2
13	Ong et al. [15]: How Does Narcolepsy Impact Health-Related Quality of Life? A Mixed-Methods Study	Behavioral Sleep Medicine	2020	Clinical Neurology	Q3
				Psychiatry	Q3
14	Barateau et al. [13]: Depression and suicidal thoughts in untreated and treated narcolepsy	Neurology	2020	Clinical Neurology	Q1
15	Francschini et al. [17]: Giving a voice to cataplectic experience: recollections from patients with narcolepsy type 1	Journal of Clinical Sleep Medicine	2020	Clinical Neurology	Q2
16	Wasling et al. [30]: Quality of life and procrastination in post-H1N1 narcolepsy, sporadic narcolepsy and idiopathic hypersomnia, a Swedich cross-sectional study.	Sleep Medicine	2020	Clinical Neurology	Q1
17	Song et al. [28]: The influential factor of narcolepsy on quality of life: compared to obstructive sleep apnea with somnolence or insomnia	Sleep and Biological Rhythms	2019	Clinical Neurology	Q4
				Neurosciences	Q4
18	Ruoff et al. [38]: High Rates of Psychiatric Comorbidity in Narcolepsy. Findings From the Burden of Narcolepsy Disease (BOND) Study of 9,312 Patients in the United States	Journal of Clinical Psychiatry	2017	Psychiatry	Q2
				Psychology- clinical	Q1*
19	Kovalska et al. [31]: Narcolepsy with cataplexy in patients aged over 60 years: a case-control study	Sleep Medicine	2016	Clinical Neurology	Q1
20	Kapella et al. [27]: Health-Related Stigma as a Determinant of Functioning in Young Adults with Narcolepsy	PloS One	2015	Multidisciplinary sciences	Q2

*Journal Citation Index 2022, WoS Web of Science, SCIE Science Citation Index Expanded

**Table 2:** Research question

		Research question
1	Kamada et al., [39]	Primary objective: to determine the burden of narcolepsy in terms of direct medical costs and comorbidities, using a health claims database. Secondary objective: to compare narcolepsy patients with patients with schizophrenia, epilepsy and ulcerative colitis as control health implications.
2	Varallo et al., [37]	To compare coping strategies amongst patients with NT1 and people without sleep disorders and to evaluate the associations between coping strategies and QoL amongst patients with NT1.
3	Bassi et al., [6]	To assess work productivity and activity impairments, and to explore their association with excessive daytime sleepiness, BMI, depression and anxiety in patients with NT1 in comparison with people without sleep disorders.
4	Li et al., [34]	To assess the impact of symptom severity on health-related quality of life in people with narcolepsy type 1. *Note: (1) differences in narcolepsy symptoms severity and health-related quality of life between drug-free and treated patients with narcolepsy type 1; (2) assess the association of selected factors (being treated, narcolepsy symptom severity) with health-related quality of life*
5	D’Alterio et al., [32]	To describe the resilience profile of narcolepsy type 1 patients and compare the results with people without narcolepsy, and to assess correlations between resilience and sociodemographic variables, narcolepsy symptoms, anxiety, depression and QoL.
6	Tadrous et al., [18]	To explore attitudes towards physical health, barriers regarding exercise and physical activity and perceptions of physiotherapy in the population of narcolepsy type 1 and 2 patients using qualitative approach od semi-structural interviews and thematic analysis.
7	Cremaschi et al., [36]	To describe the quality of life in patients with narcolepsy and the influence of the nutritional status. *Note: (1) differences in health-related quality of life between patients with narcolepsy type 1, type 2 and a control group; (2) differences in health-related quality of life between patients with narcolepsy type 1, type 2 and a control group, taking into account nutritional status, e.g., divided into normal weight, overweight and obese.*
8	Davidson et al., [16]	To investigate the impact of narcolepsy on friendship and romantic and sexual relationships.
9	Cambron-Mellot et al., [29]	To expand on the research including data from a larger number of patients, including patients with OSA and/or narcolepsy to examine the association between EDS and HRQoL to predict EQ-5D utility scores from ESS scores.
10	Chin et al., [14]	A 5-year prospective cohort study that investigates changes in QoL and symptom severity during the lifespan of NT1 and NT2 patients and to analyze predictors of long-term QoL.
11	Barateau et al., [33]	To determine the clinical, polysomnographic and biological variables associated with disrupted nocturnal sleep-in patients with narcolepsy type 1 and to evaluate the effect of medication on disrupted nocturnal sleep and its severity. *Note: (1) To compare patients with absence, mild, moderate or severe disrupted nocturnal sleep with and without medication in clinical, biological and polysomnographic characteristics; (2) To assess the effect of treatment on disruptive nocturnal sleep in a longitudinal survey (77 – 3039 days, median interval: 729.7 days)*
12	Varallo et al., [35]	To evaluate differences in the levels of addictive online behaviors between individuals with narcolepsy type 1 and sex-and age-matched healthy controls, and to evaluate the association between anxiety, depression and emotional dysregulation with problematic online gaming, problematic social media use and compulsive Internet use in individuals with narcolepsy type 1.
13	Ong et al., [15]	To identify patient-centered issues (e.g. narcolepsy symptoms) affecting health-related quality of life in people with narcolepsy and to evaluate patient-reported outcome measures using a mixed method approach.
14	Barateau et al., [13]	To assess the frequency and determinants of depressive symptoms and suicidal thoughts in adults with narcolepsy type 1 and controls as well as the changes after narcolepsy management and the risk factors of major depressive episode and suicide risk in patients with narcolepsy type 1.
15	Francschini et al., [17]	To describe how people suffering from narcolepsy frame, remember and report personal experiences with cataplexy.
16	Wasling et al., [30]	To compare subjects form Sweden on central disorders of hypersomnolence (Idiopathic hypersomnia, post-H1N1 narcolepsy, sporadic narcolepsy) and healthy controls regarding HRQoL together with symptoms of procrastination.
17	Song et al., [28]	To compare quality of life and its predictors (e.g. age, gender, BMI, severity of insomnia, EDS, anxiety, depressed mood) in patients with narcolepsy, obstructive sleep apnea and insomnia.
18	Ruoff et al., [38]	To evaluate psychiatric comorbidity patterns in patients with a narcolepsy
19	Kovalska et al., [31]	To assess health and quality of life in aged (>60 years) patients with narcolepsy comparing to age- and sex-matched controls
20	Kapella et al., [27]	To examine relationships between health-related stigma, mood and daytime functioning in young adults with narcolepsy compared to those without narcolepsy.

*BMI* body mass index

**Table 3:** Target population, sample, recruitment

		Region	Data collection	Sample: inclusive/exclusive criteria, recruitment	Groups
**1**	Kamada et al., [39]	Japan	2017–2022	**Inclusion:** Data for this research were conducted from a database by JMDC Inc. Data were extracted in September 2022. Selection: a) diagnosed with narcolepsy according to the ICD-10, b) aged 18 years and older, c) enrolling continuously for at least 1 year. **CG**: a) diagnosed with control disease at least twice (ICD-10), b) and c) are the same as for narcolepsy.	**NT1/2** n=4 594 Male: n=2 695 (58.7%) Mean age/SD: 35.2/12.7 **SCHI** n=18 376 Male: n=10 780 (58.7%) Mean age/SD: 35.8/12.0 **EPI** n=18 376 Male: n=10 780 (58.7%) Mean age/SD: 35.8/12.2 **UCL** n=4 594 Male: n=2 695 (58.7%) Mean age/SD: 36.7/11.2
**2**	Varallo et al., [37]	Italy	2017–2019	**Inclusion: a)** age of participants over 18, b) NT1 according to ICSD-3, c) ability to understand the purpose of the study, d) ability to read Italian **CG:** matching narcolepsy group by age and sex **Recruitment:** Narcolepsy Center of Bologna, Italy; CG was recruited by family members of patients or companions of patients.	**NT1** n=122 Male: n=59 (48.4%) Mean age/SD: 37.5/15.3 **CG** n=138 Male: n=59 (42.7%) Mean age/SD: 39.5/14.1
**3**	Bassi et al., [6]	Italy	Not specified	**Inclusion:** patients with diagnosed NT1 according to the ICSD-3. Must have been over 18 years of age. **CG:** matching NT1 group but does not have a sleep disorder diagnosis. Must have been over 18 years of age. **Recruitment**: Narcolepsy Centre of Bologna, Italy.	**NT1:** n=127 Male: n=59 (46.5%) Mean age/SD: 38.2/15.5 **CG:** n=131 Male: n=53 (40.5%) Mean age/SD: 37.4/14.3
**4**	Li et al., [34]	China	2019 –2021	**Inclusion:** visited sleep clinic in period between September 2019 to February 2021, aged more than 18 years and diagnosed with NT1 **Exclusion:** comorbidity of other sleep disorder or clinically unstable medical condition **Recruitment:** visitors of a sleep clinic at Peking University People’s Hospital, regular follow-up	**NT1** n=174 Male: n=119 (68.4%) Mean age/SD: 31.14/11.28
**5**	D’Alterio et al., [32]	Italy	2017–2019	**Inclusion:** patients with NT1 according to the ICSD. Participants had to be over 18 years of age. **CG:** participants without sleep disorders and over 18 years of age. **Recruitment:** Narcolepsy Centre of Bologna, Italy. CG was recruited from those accompanying patients at the tertiary neurological outpatient clinic of the IRCCS Institute of Neurological Science of Bologna (ISNB).	**NT1** n=137 Male: n=65 (52.6%) Mean age/SD: 38.0/15.6 **CG** n=149 Male: n=66 (44.3%) Mean age/SD: 39.6/14.0
**6**	Tadrous et al., [18]	Ireland	Not stated	**Inclusion:** a) being between 18 – 65 years of age, b) diagnosed with NT1 or NT2 according to the ICSD-3 for at least 6 months, c) had to understand English, d) sign written consent. **Exclusion**: other sleep disorders than NT1/2. **Recruitment:** St. James’s Hospital, Dublin, The Narcolepsy Clinic – National Narcolepsy Centre. Patients were interviewed after the appointment at the specialist, they were informed 5–7 days before.	**NT1/2** n=22 Male: n=10 (45.45%) Mean age/SD: 31.4/13.2
**7**	Cremaschi et al., [36]	Brazil	2015 –2016	**Inclusion:** Adult patients of a Sleep Center from Sao Paulo-Brazil with diagnosed narcolepsy **CG:** students and employees of the University with a normal Epworth sleepiness scale and Stop-Bang questionnaire score **Recruitment:** Patients of a Sleep Center from Sao Paulo-Brazil recruited during regular follow up in an outpatient clinic * Divided based on BMI on normal weigh, overweight, obesity	**NT1** n=33 Male: n=6 (18.2%) Mean age/SD: 36.2/10.2 **NT2** n=33 Male: n=6 (18.2%) Mean age/SD: 36.2/10.2 **CG** n=33 Male: n=6 (18.2%) Mean age/SD: 36.3/9.7
**8**	Davidson et al., [16]	US	2020	**Inclusion:** young adults aged 18–39 years, lived in US, fluent in English, were diagnosed with narcolepsy NT1 or NT2 **Exclusion:** uncompleted questionnaire/n=87), incorrect reports (n=9) **Recruitment:** Recruited through national narcolepsy patient organizations. E-mails with link on **anonymous online survey** sent by 5 organizations to their members.	**NT1 or NT2** n=254 Male: n=32 (12.6%) Mean age/SD: 28.8/5.6
**9**	Cambron-Mellot et al., [29]	UK	2016–2017	**Inclusion:** Participants had to be over the age of 18 and the data were nation-based using quota for age and sex. Respondents were selected if they were patients with OSA and/or narcolepsy. **Recruitment:** data were available from 5 European countries (France, Germany, the UK, Italy and Spain) from the national Health and Wellness Survey – internet/telephone based self-administrative.	**NT w/o OSA** n=48 Male: n=23 (47.9%) Mean age/SD: 49.0/17.8 **NT w OSA** n=23 Male: n=16 (69.6%) Mean age/SD: 53.3/14.1 **OSA w/o NT** n=2 277 Male: n=1 606 (70.5%) Mean age/SD: 59.3/12.5
**10**	Chin et al., [14]	Taiwan	2013–2019	**Inclusion:** Respondents had to be newly diagnosed with NT1 or 2 according to the ICSD-3. They had to be aged between 16 to 45 years and did not receive medication for narcolepsy before. **Exclusion**: another sleep disorder, other neurological diseases a patient had, severe cardiovascular disease history, intellectual disability history, patients with shift work or circadian rhythm disorders.	**NT1/2** n=157 NT1=111, NT2 =46 Male: n=89 (56.6%) Mean age/SD: 23.9/8.73
**11**	Barateau et al., [33]	France	2014 –2020	**Inclusion:** adult patients with NT1 monitored by the National Reference Center for Narcolepsy, Montpellier, France, full polysomnographic data, no missing data on NSS **Recruitment:** National Reference Center for Narcolepsy, Montpellier, France * Divided into drug-free (n=145) and treated (n=103) patients. Among drug-free patients, 51 were re-evaluated under stable treatment after 77–3039 days.	**NT1** n=248 Male: n=137 (55.2%) Mean age/SD: 39.03/15.86
**12**	Varallo et al., [35]	Italy	2020	**Recruitment:** Recruited through the Italian Association of Patients with Narcolepsy and Hypersomnia via an **anonymous online survey** published on a website. **CG:** acquaintances: participants identify CG among friends and/or family members	**NT1** n=43 Male: n=36 (42.0%) Mean age/SD: 32.21/10.43 **CG** n=86 Male: n=36 (42.0%) Mean age/SD: 32.02/10.18
**13**	Ong et al., [15]	US	2018	**Inclusion:** at least 18 years old, report elevated symptoms of depression (PHQ-9 score over 10), have documented diagnosis of narcolepsy, stable Internet connection, fluent in English **Recruitment:** Recruitment through various online resources. Those who expressed interest (n=75) get e-mail with link to fill in a questionnaire and those who met criteria for the study (n=64) were scheduled for a brief phone screening and received a link for a full questionnaire. From those who were eligible to continue and provided full documentation (n=30) were invited for a focus group.	**NT1 or NT2** n=29 Male: n=2 (6.9%) Mean age/SD: 31.07/ 7.57
**14**	Barateau et al., [13]	France	2009 – 2015	**Recruitment:** National Reference Center for Narcolepsy, Montpellier, France **CG:** recruited from the general population via advertisement and local association networks.	**NT1** n=297 Male: n=172 (58.0%) Mean age/SD: 38.90/17.30 **CG** n=346 Male: n=173 (50.0%) Mean age/SD: 37.57/16.00
**15**	Francschini et al., [17]	Italy	2017	**Recruitment:** Recruited through outpatient clinic for narcolepsy, Narcolepsy Center of the University Bologna	**NT1** drug naive n=22 Male: n=12 (54.5%) Mean age/SD: 46.00/16.56
**16**	Wasling et al., [30]	Sweden	2019–2020	**Inclusion:** patients had to be diagnosed according to ICSD-3 and to be 18 to 65 years of age. **Recruitment:** patients of the Department of Neurology, Sahlgrenska University Hospital. **CG:** recruited from the local community, inclusion was the ability to understand and provide written consents and could not have any neurological or psychiatric disease with no current medication for sleep or cognition.	**Post-H1N1 NT1** n=24 Male: n=22 (48.9%) Mean age/SD: 27.3/8.4 **NT1 sporadic** n=23 Male: n=9 (39.1%) Mean age/SD: 41.6/11.1 **IH** n=21 Male: n=6 (28.6%) Mean age/SD: 43.00/15.3 **CG** n=23 Male: n=10 (43.5%) Mean age/SD: 36.6/12.0
**17**	Song et al., [28]	Korea	2011 –2016	**Recruitment:** Retrospectively screened patients who visited a tertiary sleep center	**NT1 or NT2** n=63 Male: n=43 (68.3%) Mean age/SD: 27.03/9.29 **OSA** n=49 Male: n=43 (87.8%) Mean age/SD: 39.20/11.83 **INS** n=87 Male: n=32 (36.8%) Mean age/SD: 47.25/13.05
**18**	Ruoff et al., [38]	US	2006 – 2010	**Inclusion:** at least 18 years old. Data were collected from The Truven Health Analytics MarketScan Research Databases.	**NT1/2** N=9 312 Male: n=3 799 (40.7%) Mean age/SD: 46.1 (13.3) **CG** N=46 559 Male: n=18 995 (40.8%) Mean age/SD: 46.1 (13.3)
**19**	Kovalska et al., [31]	Czech Republic	2012 – 2015	**Inclusion**: invitation of patients over 60 who meet ICSD-2 criteria for narcolepsy with undisputable cataplexy **Recruitment**: identification of patients in departmental database of a sleep clinic and according to the regular frequency of clinic attendance. **CG:** age and sex-matched control group recruited from a leisure activity center for the elderly (University of Third Age)	**NT1** n = 42 Male: n=18 (42.9%) Mean age/SD: 71.9/7.5 **CG** n=46 Male: n=19 (41.3%) Mean age/SD: 72.2/7.0
**20**	Kapella et al., [27]	US	2002	**Inclusion:** young adults (age 18–35) with narcolepsy This study utilized data collected by Merritt et al. (2004). **NT1 or NT2:** Patients with narcolepsy who contacted the Center for Narcolepsy Research, University of Illinois at Chicago, and indicating interest in participating in research. **CG**: acquaintances Respondents were mailed a packed that included questionnaires and self-addressed postage paid envelope.	**NT1 or NT2** n=122 Male: n=27 (22.1%) Mean age/SD: 27.1/5.0 **CG** n=93 Male: n=34 (36.6) Mean age/SD: 25.7/4.0

NT1 – group of patients with narcolepsy type 1; NT2 – group of patients with narcolepsy type 2; CG – age- and sex- matched control; OSA – group of patients with obstructive sleep apnea; INS – group of patients with somnolence or insomnia, IH – idiopathic hypersomnolence patients, SCH – schizophrenic patients, UCL – ulcerative colitis patients, EPI – group of patients with epilepsy

**Table 4:** Design, concepts, measures

	AUTHORS / YEAR	DESIGN	CONCEPTS / MEASURES			
			Sociodemographic, anamnestic data	Sleepiness symptoms and sleep quality	Other concepts	QoL
1	Kamada et al., [39]	quantitative retrospective, routine database-based	years of observation, Insurance type, Admission, Inpatient days, Outpatient visits,		Physical, psychiatric and psychological comorbidities	
2	Varallo et al., [37]	quantitative questionnaire-based cross-sectional study	education, working, age of onset and age of diagnosis, pharmacotherapy		Coping: COPE	EQ-5D
3	Bassi et al., [6]	quantitative questionnaire-based cross-sectional study	education, had a partner, occupational status, weekly work hours and schedule, shift work, disabled	ESS	Depression: BDI Anxiety: STAI Work productivity: WPAI	
4	Li et al., [34]	quantitative questionnaire-based cross-sectional study	education, age of onset, disease duration, diagnosis delay, polysomnography measurements	NSS	Nutrition status: BMI	EQ-5D
5	D’Alterio et al., [32]	quantitative questionnaire-based cross-sectional study	education, had a partner, student or employment, BMI	ESS	Anxiety: STAI Depression: BDI Resilience: RS	SF36
6	Tadrous et al., [18]	qualitative semi-structural study interviews	living arrangements, highest educational level, current education/employment status, medication			Open-ended questions
7	Cremaschi et al., [36]	quantitative questionnaire-based cross-sectional study	ethnicity, education, occupation, comorbidity, medication	ESS	Depression: BDI Nutrition status: BMI	SF36
8	Davidson et al., [16]	quantitative questionnaire-based cross-sectional study enriched by open-ended questions	race, employment, age of onset, age at diagnosis, medication		Social support: MSPSS Social and romantic relationships Communication with other regarding narcolepsy	
9	Cambron-Mellott et al., [29]	quantitative retrospective, routine database-based	country, marital status, university degree, household income, BMI, smoking, alcohol use, exercise	ESS		EQ-5D
10	Chin et al., [14]	quantitative questionnaire-based longitudinal study	Age of onset, BMI, NT symptoms occurrence,	SSI ESS		SF36
11	Barateau et al., [33]	quantitative questionnaire-based cross-sectional study	medical interview: age at onset, age at diagnosis, disease duration, cataplexy frequency, comorbidity, polysomnography measurements	ESS NSS ISI	Depression: BDI Anxiety: STAI Autonomic dysfunction: SCOPA-AUT	EQ-5D
12	Varallo et al., [35]	quantitative questionnaire-based cross-sectional study	marital status, educational level, occupation medication,	ESS NSS	Depression, anxiety: DERS, DASS-21 Addictive online behavior: IGDS, CIUS, BSMAS	
13	Ong et al., [15]	mixed method cross-sectional study questionnaire-based study focus groups	race, educational level time since diagnosis	ESS	Depression: PHQ-9	SF36 PROMIS
14	Barateau et al., [13]	quantitative cross-sectional study questionnaire-based and longitudinal (treatment effect)	education level polysomnography measurements	ESS NSS ISI Cataplexy Frequency Scale	Depression: BDI, MINI Nutrition status: BMI Autonomic dysfunction: SCOPA-AUT	EQ-5D
15	Francschini et al., [17]	qualitative semi-structural study interviews	disease onset, medication			Narrative inquiry approach
16	Wasling et al., [30]	quantitative cohort study questionnaire-based	Disease duration, BMI, orexin levels, sleep latency test, medication	ESS	Health: PHQ-9 Fatigue: FSS Procrastination: PPS, IPS, STS	EQ-5D-5L
17	Song et al., [28]	quantitative cross-sectional study questionnaire-based	polysomnography measurements	ESS ISI	Depression, anxiety: HADS Nutrition status: BMI	SF36
18	Ruoff et al., [38]	quantitative retrospective, routine databases-based	geographic region, health insurance status		Psychiatric comorbidity	
19	Kovalska et al., [31]	quantitative cross-sectional study questionnaire-based	marital status, family status, education, employment changes, disability pension, retirement pension, work after retirement, length of economic inactivity medication, comorbidity	ESS	Depression, anxiety: GDS, STAI Cognitive functioning: ACE Physical fitness: SPPB Nutrition status: BMI	EQ-5D
20	Kapella et al., [27]	quantitative cross-sectional study questionnaire-based	race, education, economic status, marital status, employment status	ESS PSQI FOSQ	Depression, anxiety: HADS Health-related stigma: SSIS, DCS	SF36

*BMI *body mass index; *EQ-5D *european quality of life-5 dimensions questionnaire [23]; *NSS *narcolepsy severity scale [20]; *BDI *beck depression inventory [25]; *HADS *hospital anxiety and depression scale [26]; *MSPSS *multidimensional scale of perceived social support [40]; *STAI *stanford trait anxiety inventory [41]; *GDS *geriatric depression scale [22]; *IGDS *internet gaming disorder scale [43]; *CIUS *compulsive internet use scale [44]; *BSMAS *bergen social media addiction scale [45]; *DERS *difficulties in emotion regulation scale [46]; *DASS*−21 depression anxiety and stress scale [47]; *PHQ*−9 patient health questionnaire [48]; *PROMIS *depression, anxiety, fatigue, sleep disturbances, sleep-related impairment, pain interference, physical functioning [24]; *MINI *international neuropsychiatric interview [49]; *SCOPA-AUT *scale for outcomes in parkinson’s disease-autonomic dysfunction [50]; *ISI *insomnia severity index [21]; *ACE *addenbrook cognitive examination [51]; *SPPB *short physical performance battery [52]; *PSQI *pittsburgh sleep quality index [53]; *FOSQ *functional outcomes of sleep questionnaire [54]; *SSIS *stigma and social impact scale [55]; *DCS *disclosure concerns scale [56]; *ESS *epworth sleepiness scale [19]; *SF*−36 short form health survey [22], *COPE * the coping orientation to problems experienced inventory [57]; *WPAI *the work productivity and activity impairment questionnaire – general health [58]; *RS *resilience scale – Italian version [59]; *SSI *the stanford sleep inventory [60]; *FSS *fatigue severity scale [61]; *PPS*(12) pure procrastination scale [62]; *IPS *the irrational procrastination scale [63].

### What is it like to live with narcolepsy based on published studies?

According to the research articles presented, living with narcolepsy causes health-related impairments and lower QoL while compared with people without narcolepsy or people with other diseases. The differences and effects are usually visible when comparing people with and without narcolepsy, comparing patients according to symptom severity, comparing NT1 and NT2 patients and considering treated/not-treated, etc.

Song et al. [28] compared QoL factors in narcolepsy patients to those with obstructive sleep apnea (OSA) and insomnia. Narcolepsy patients had significantly lower insomnia rates than insomnia patients, but higher EDS symptoms compared to both insomnia and OSA groups. No differences were observed in anxiety, depression, or overall QoL between the groups, nor between NT1 and NT2 subtypes. Depressive mood and anxiety were identified as predictors of QoL. Cambron-Mellott et al. [29] studied the relationship between excessive daytime sleepiness (EDS) and HRQoL in patients with OSA, with or without narcolepsy, using ESS scores. A significant breakpoint at an ESS score of 12 indicated sleep impairment, with higher ESS scores correlating to lower HRQoL. While HRQoL did not differ between patients with OSA or narcolepsy alone, those with both conditions had significantly lower scores.

Wasling et al. [30] examined HRQoL and procrastination in patients with central hypersomnolence, finding greater EDS, anxiety, and depression in post-H1N1 NT1 patients compared to sporadic NT1 patients. Procrastination was notably higher in the post-H1N1 group, associated with task aversiveness, distant deadlines, and low self-control.

Kovalska et al. [31] conducted a case-control study on elderly Czech narcolepsy patients, finding they smoked more (17.9 cigarettes per day) compared to the controls (4.9 cigarettes per day) on average.. Cognitive complaints, such as memory, concentration, and attention impairments, were frequent but not statistically significant. Narcolepsy patients exhibited significantly lower QoL compared to controls.

D’Alterio et al. [32] explored resilience in NT1 patients, revealing significantly lower resilience levels compared to controls, with resilience declining with age. Patients with higher resilience reported lower anxiety and depression levels and had QoL scores comparable to healthy controls, highlighting resilience as a key factor in QoL.

Sociodemographic analyses showed no differences between age, gender and level of education in most of the explored studies [13, 33–35].

### Symptom severity and QoL in narcolepsy

The symptomatology of narcolepsy and its severity has an impact on overall HRQoL. Li et al. [34] found strong correlations between symptom severity, especially excessive daytime sleepiness (EDS), cataplexy and sleep disruption with self-care, usual activities, pain and discomfort, anxiety and depression. Ong et al. [15] used focus groups to find areas of impairment and challenges in people with narcolepsy. They found the constancy of EDS symptom was a key factor of having a poor HRQoL.

Poorer HRQoL was found in patients with NT1 (with cataplexy) vs patients with NT2 patients. Cremaschi et al. [36] found that all domains of SF-36 [22] were lower in narcolepsy patients in comparison with a control group, with the most affected being Role-physical, Role-emotional and Energy/Fatigue. Role-physical was the only one to differ in between NT1 and NT2, being worse in NT1 patients. Poorer general health in NT1 vs NT2 patients was confirmed by Ong et al. [15]. Bassi et al. [6] evaluated work activity and productivity impairments in NT1 patients compared to healthy controls, focusing on connections with EDS and other indicators. Even treated NT1 patients were more likely to be classified as disabled, with activity and productivity impairments three times greater than controls. Sleepiness was linked to activity impairments, while depression and anxiety were tied to work and non-work impairments in both groups. Nearly all NT1 patients experienced difficulties in job effectiveness and daily activities despite treatment.

Moreover, narcolepsy patients with cataplexy (NT1) usually suffer with the burden of narcolepsy symptoms more than patients with NT2. This is suggested in the study of Franceschini et al. [17], who used a qualitative approach to describe experiences with cataplexy in patients with NT1. These experiences were connected in three clusters: 1) trigger of cataplexy; 2) bodily sensations while experiencing cataplexy; 3) management and control strategies (blocking or controlling emotional expressions). On the other hands, Tadrous et al. [18] conducted qualitative research on perceptions of physical health, physiotherapy, and barriers to exercise. They identified three main themes. First, barriers to exercise included psychological factors (e.g., anxiety), fatigue, fear of cataplexy, and accessibility issues (timing, cost). Motivators for exercise were career and educational goals, as well as social and health well-being. However, patients prioritized social aspects, like career, family, and relationships, over physical activities. Physical health concerns included weight gain, reduced activity, and pain. The role of physiotherapy was highlighted in providing guidance, exercise programs, and external motivation to support adherence.

Chin et al. [14] examined health-related quality of life (HRQoL) changes (using SF-36) and symptom severity in narcolepsy patients over five years. Physical domains remained stable, with physical role functioning and general health initially scoring low; however, physical role functioning improved over time, particularly in the NT1 group. Based on these findings, the authors suggest implementing exercise programs for narcolepsy patients. Significant improvements were observed in social and emotional roles following treatment onset, though vitality and psychological health remained unchanged. The NT2 group had higher initial scores across SF-36 domains than NT1, but after five years, NT2 patients showed declines in all domains, with slight improvement only in emotional role functioning.

### Impact of NS on relationships and mental health

NT1 patients face significant psychosocial challenges, including anxiety about cataplexy in social settings. Davidson et al. [16] found that 98.4% of young adults with narcolepsy reported difficulties in their social life, with nearly 50% stating they had few friends. Regarding romantic relationships, almost 90% said narcolepsy hindered forming or maintaining relationships, while over 80% noted its impact on their sexual life. Around one-third experienced cataplexy during sex, and half reported falling asleep during sexual activity. Perceived social support from friends and family did not significantly differ between NT1 and NT2 patients.

Kapella et al. [27] highlighted health-related stigma as a key factor affecting functioning and HRQoL in young adults with narcolepsy. Compared to their peers without narcolepsy, these patients reported worse mood, lower HRQoL, and greater stigma, which indirectly contributed to depressive symptoms. These findings align with stigma-related outcomes observed in other chronic illnesses such as epilepsy, multiple sclerosis, and HIV/AIDS.

Varallo et al. [35] examined addictive behaviors, finding that NT1 patients exhibited higher rates of problematic online gaming compared to controls, though no differences were observed in general Internet use or social media habits. These social impairments may contribute to broader behavioral challenges. Varallo et al. [37] investigated coping strategies finding significantly lower HRQoL in the narcolepsy group compared with controls. These patients exhibited specific coping patterns, such as reduced use of active coping, planning, and suppression of competing activities, alongside increased use of mental and behavioral disengagement, including avoidance and distancing.

Ruoff et al. [38] analyzed routine database data and found higher psychiatric comorbidity in narcolepsy patients compared to controls, with elevated anxiety levels particularly in young adults. Narcolepsy patients also showed greater reliance on psychiatric medications, both for narcolepsy treatment and at higher rates than controls with similar conditions. Similarly, Cremaschi et al. [36] reported higher antidepressant use among narcolepsy patients. Although psychiatric drugs are often prescribed for cataplexy in NT1, the authors noted that their use remains disproportionately high even when accounting for this.

### The effect of treatment on narcolepsy symptoms and HRQoL

Comparing treated and untreated patients helps clarify the impact of medication on narcolepsy symptoms and QoL. Untreated patients often experience more severe symptoms and poorer QoL than those on treatment. For instance, Barateau et al. [33] reported that over half of untreated patients and nearly 40% of treated patients had moderate to severe sleep disruption symptoms, increased EDS, anxiety, depressive symptoms, and poor QoL. Polysomnography differences were notable: treated patients showed impairment mainly in nocturnal REM sleep, while untreated patients exhibited issues in sleep efficiency, total sleep time, sleep attacks, sleep-wake transitions, sleep instability in REM and non-REM sleep, NT1 percentage, and WASO. Sleep disruption was common in NT1 patients, linked to greater disease burden and poorer QoL. Li et al. [34] found that treatment improved HRQoL and reduced symptom severity.

Kamada et al. [39] found that narcolepsy patients had annual medical costs three times higher than the general population, driven by frequent outpatient visits for medication management. Comorbidity rates were like schizophrenia, but narcolepsy patients experienced more mental and behavioral issues than those with epilepsy or ulcerative colitis. Barateau et al. [13] found high depression scores in narcolepsy patients, especially NT1, with untreated patients reporting more suicidal thoughts, linked to factors like obesity, severe symptoms, and lower education. Treatment improved HRQoL, reduced symptoms, and lowered suicidal thoughts, with higher risks seen in males and those with frequent cataplexy, parasomnias, or high ESS [19], ISI [21], and NSS [20] scores.

### Gaps in the body of knowledge, or what do we need to explore?

The analyzed data were presented to stakeholders (medical doctors) who were asked to assess them and determine gaps, possibilities and directions for future research. The following recommendations were formulated:

### Somatic and mental comorbidities with a focus on knowledge

1. The considerable impact of somatic and mental comorbidities on QoL as well as diagnostic delay indicate a need to disseminate knowledge beyond physicians and neurology specialists to the general public.

### Psychosocial aspects and adherence to treatment

2. While the burden of symptoms and poor mental health in narcolepsy patients was explored, little is known about the social and emotional aspects of living with narcolepsy or about the impact of narcolepsy on one’s personality (e.g., narcoleptic personality traits changes).

3. Focus on coping strategies to handle living with narcolepsy (e.g. strategies of wakefulness or cataplexy management), or the role of community support regarding the restrictions in participation of narcolepsy patients should be addressed in future research.

4. The effect of pharmacological as well as non-pharmacological treatment (e.g. daily routine, flexible working hours) as management of symptom severity and its impact on QoL and challenges of adherence were not addressed sufficiently in the searched literature.

5. Screening for excessive daytime sleepiness in the general population could be helpful in identifying new patients with narcolepsy, shortening the time between symptom onset and disease diagnostics and initiating treatment, which could lead to overall QoL improvement.

### Sample and recruitment strategies

6. In screened research, the recruitment of patients via sleep clinics or patients’ organizations was quite successful. But only rarely were newly diagnosed patients and younger/older narcolepsy patients the focus of research.

### Lack of qualitative studies

7. The strength of the existing research is the utilization of comparable measurements and methodological strategies using cross-sectional quantitative and questionnaire-based methods. However, more explorative, qualitative studies are needed to identify facilitators and barriers to promote high QoL while living with narcolepsy.

## Discussion

In this study, we aimed at a scoping review of peer-reviewed articles published between 2014–2025 by searching the Web of Science and PubMed databases to map and synthesize evidence about Quality of Life (QoL) in narcolepsy patients, with a focus on their origin, research questions, methods and materials, participants and results.

There is an increasing trend in studies focused on QoL in narcolepsy patients, but its spread is very limited across various audiences. Most papers were published in the Clinical Neurology, Psychiatry or Neurosciences category of journals. While increasing numbers of publications might contribute to better awareness and a shortening of diagnosis delay, the narrow scope of the audience might prevent spreading knowledge beyond the neurology field, where it is needed the most. Better awareness about this rare illness, its symptoms and impact on QoL among health care professionals outside the neurology field might be essential for earlier identification of symptomatology occurring in people who may later be underdiagnosed or misdiagnosed. The average time of diagnostic delay is around 10 years from the first symptom manifestation to being diagnosed [5]. This occurs mostly in the teenage years, which is the most common time of first symptomatology manifestation, in some cases with 20 years delay of diagnosis. They also suggest that the presence of cataplexy facilitates and accelerates the correct diagnosis, i.e. diagnostic delay is longer in patients in whom this symptom is either not present or has not been asked for by treating physicians [64]. Conversely, the diagnosis of narcolepsy is highly inconsistent across different sleep clinics and narcolepsy centers, and accurately diagnosing the condition—whether type 1 or type 2—remains a significant challenge. The findings presented in Table 3 highlight the diverse array of methods and diagnostic criteria employed, including various guidelines and manuals. This variability fosters a wide range of diagnostic approaches, leading to inconsistencies in diagnostic practices. Such heterogeneity has the potential to adversely affect the reliability and comparability of studies investigating narcolepsy and its impact on HRQoL.

Most of the studies in this review assess the association of narcolepsy symptoms, treatment, mental health or nutritional status with QoL in patients with narcolepsy, and only one study focused on social relationship, and two studies were exploratory focusing on personal experiences with cataplexy and attitudes towards physical activity. Most of the studies were quantitative, comparing control groups with narcolepsy patients, and used a similar or the same questionnaire-based approach. Frequently used instruments for measuring QoL were the SF-36 [22] and the EQ-5D [23]. Sleepiness-related indicators were measured mostly using the ESS [19] and the NSS [20]. The use of these instrument can, for example, be helpful in research articles comparing data. On the other hand, quality of life as a construct is described by authors differently, and the whole concept of measuring with questionnaires does not always endorse the nature of subjectively perceived life quality by patients. Therefore, the lack of qualitative studies in our sample points out the need for qualitative approaches while describing HRQoL through patients’ perspective. Our scoping review worked with only two qualitative studies [17, 18] focused specifically on the perception of cataplexy and physical activity. One study used mixed method research [15] using basic thematic analysis [65]. In the additional search after the primary screening for articles we found one study focused on a qualitative approach with the broader aim of narcolepsy impact. Schokman et al. [66] did 24 interviews focusing on understanding the patients’ perceptions of narcolepsy, dividing their findings into five thematic clusters: understanding experiences of narcolepsy; understanding the impact on daily life, identity perception and narcolepsy labeling; understanding narcolepsy management and long-term care; and the perception of narcolepsy as a disorder.

Narcolepsy patients were recruited in the studies using regular follow-up at sleep clinics or via national reference centers, but patients’ organizations were also employed to conduct a study. No study focused on identifying new patients and their QoL. Together with diagnostic delay, the emerging necessity of EDS and/or narcolepsy populational screening seem to be a solution to identify new patients and to look at their QoL, understanding the differences between diagnosed and undiagnosed patients; the difficulties with suffering from rare disease could also be helpful to closer describe QoL apart from questionnaires. As Bryson et al. [67] suggest, living with a rare disease can lead to difficulties in disease management, activity limitations, diagnostics and treatment process. Psychologically, the effect of uncertainty, distress, guilt, etc. could lead to worse QoL. Receiving instrumental support, the involvement of insurance companies, financial aid or distress could play a crucial role. The social aspects of rare diseases could affect QoL through the lack of social participation, social support, informational support or stigma.

Living with narcolepsy is associated with worse QoL, poor mental health, higher psychiatric comorbidity and symptoms of narcolepsy, and its severity plays a significant role in these associations. People who do not take medication show more severe symptoms or poorer QoL compared with treated patients. The perception of disease can differ according to multiple factors, e.g., age of onset or diagnostic delay. Only one study in our review focused specifically on elderly patients [31] and one on young adults [27]. In the study of Zhang et al. [68], a comparison between adult and child patients with narcolepsy, differences in clinical characteristics of the overall measures were presented (children tend to be more obese and were more likely to eat during night, showed an increase in sleep drunkenness and more parasomnia experiences). Therefore, the necessity for more research papers based on age and age of onset would be helpful, and for setting a better treatment plan, both pharmacological but also non-pharmacological, which is a great part of the symptom’s severity management.

According to Barateau et al. [69], non-pharmacological treatment strategies are important for a patient’s disease management due to the dangerousness of symptom manifestation. Bassetti et al. [70] suggest non-pharmacological treatment should be the first considered. Apart from the usually advised management strategies, authors also recommend that patients join a patients’ organization, which can help not only with social support and understanding, but also with providing information and arranging (non)pharmacological treatment strategies. This could be important for those patients who tend to stay drug-free [34, 13, 33]. The necessity of information about this disease seems to be crucial not only for management of the symptoms, but also for the public, to shorten the diagnostic delay; and for the population of specialists (psychologists and psychotherapists, nurses, physiotherapists, etc.) who play a significant role in narcolepsy treatment and management.

## Strengths and limitations

Our study employed scoping review methodology, guided by the PRISMA-ScR [11] statement and Mak & Thomas’s approach [12], to assess the depth of existing literature on narcolepsy’s impact on quality of life (QoL) and identify methodological gaps in the field. We also incorporated insights from medical experts, psychologists and researchers to complement our findings and provide recommendations for future research. Unlike previous reviews (e.g., Tadrous et al. [18], Raggi et al. [3]), our study highlights both the current state of research methodologies and clinicians’ perspectives. Limitations include reliance on a limited number of databases (WoS, PubMed) and strict inclusion criteria (e.g., English-only, post-2014 studies), which may have excluded relevant studies. The enriching content of stakeholders’ consultation we see a limitation in the low number of stakeholders contacted. Despite this, our review establishes a foundation for further research and comparative studies together with future possibilities and suggestions from the experts in the field.

## Conclusion

This study aimed to provide a scoping review of twenty original articles examining quality of life in patients with narcolepsy. We focused on scoping the research questions, method, participation and recruitment in order to understand the nature of research in this field and to determine gaps and possibilities with a team of stakeholders. Future directions for research may be possible in more qualitative work, in screening methods focused on finding new patients, spreading information among physicians, specialists and the general population, the psychological and somatic aspects of the disease and adherence to treatment, and focusing on patients through their age groups.

## Supplementary Information

Below is the link to the electronic supplementary material.

Supplementary file1: PRISMA checklist 1 (DOCX 26.0 KB)

Supplementary file2: PRISMA checklist 2 (DOCX 108 KB)

## Data Availability

My manuscript has no associated data.

## Appendix

### Search strategy for PubMed

1. Search: narcolepsy[Title] Filters: Full Text
2. Search: (narcolepsy[Title] AND (fft[Filter])) AND (english[Language]) Filters: Full text
3. Search: ((narcolepsy[Title] AND (fft[Filter])) AND (english[Language]) AND (fft[Filter])) AND ((“2014/01/01”[Date-Publication] : “2025/12/31”[Date-Publication])) Filters: Full text
4. Search: (child) OR (children) Filters: Full text
5. #Search: ((narcolepsy[Title] AND (fft[Filter])) AND (english[Language]) AND (fft[Filter])) AND ((“2014/01/01”[Date-Publication] : “2025/12/31”[Date-Publication])) AND (fft[Filter])) NOT ((child) OR (children) AND (fft[Filter])) Filters: Full text
6. Search: ((quality of life) OR (health-related quality of life)) OR (mental health) Filters: Full Text
7. Search: ((narcolepsy[Title] AND (fft[Filter])) AND (english[Language]) AND (fft[Filter])) AND ((“2014/01/01”[Date-Publication] : “2025/12/31”[Date-Publication])) AND (fft[Filter])) NOT ((child) OR (children) AND (fft[Filter])) AND (fft[Filter])) AND (((quality of life) OR (health-related quality of life)) OR (mental health) AND (fft[Filter])) Filters: Full text
